# Social Media Addiction and its Associated Factors Among High School Adolescents in Türkiye

**DOI:** 10.1007/s10900-026-01578-7

**Published:** 2026-04-20

**Authors:** Sibel Sert, Alaettin Ünsal, Remziye Can

**Affiliations:** 1 Department of Public Health, Eskisehir Osmangazi University, Eskisehir, Türkiye; 2 Mustafa Kemal Atatürk Vocational and Technical High School, Eskisehir, Türkiye

**Keywords:** Social media addiction, Subjective happiness, Adolescents, Mental health

## Abstract

The widespread use of social media among adolescents has raised concerns about social media addiction and its potential impact on psychological well-being. This study aimed to examine social media addiction and its associated factors, including subjective happiness, among high school students. This cross-sectional study was conducted between February and April 2025 among 858 high school students using a cluster sampling design. Data were collected through a self-administered questionnaire including the Social Media Addiction Scale for Adolescents (SMAS-A) and the Subjective Happiness Scale. Non-parametric tests, multiple linear regression analysis, and Spearman correlation analysis were performed. The mean SMAS-A score was 20.8 ± 7.1, indicating a moderate level of social media addiction. Multiple linear regression analysis showed that earlier age of social media initiation, longer daily social media use, poorer friendship relationships, and lower subjective happiness were significant predictors of higher social media addiction scores, whereas female gender was associated with lower addiction levels. A weak but significant negative correlation was observed between social media addiction and subjective happiness. In conclusion, social media addiction among high school students was influenced by both behavioural and psychosocial factors. Interventions addressing excessive social media use and supporting adolescents’ well-being may help reduce addiction risk.

## Introduction

In recent years, the rapid advancement of digital technologies has led to a substantial increase in social media use both in Türkiye and worldwide. Continuously evolving and increasingly widespread social media platforms have become essential tools that individuals rely on for learning, communication, and accessing information. According to the Global Digital Report [1], as of January 2024, approximately 5.04 billion people were active social media users globally. The Turkish Statistical Institute [2] reported that 88.8% of individuals in Türkiye use the internet, with usage rates of 92.2% among men and 85.4% among women. Among those aged 16–74 years, 87.1% use the internet, and 66.1% of children are social media users.

Young people spend a considerable amount of their time on social media due to its ability to provide easy and immediate access to various activities such as entertainment, visual sharing, and real-time communication [3]. While social media use offers many conveniences, it may also lead to problematic use among adolescents, including addiction-like behaviours and an increased tendency to access mobile social media while walking, shopping, or performing other activities [4, 5].

Social media addiction is defined as excessive use of social media, a persistent desire to check social media accounts, an inability to stop using social media despite attempts to do so, and feelings of tension or irritability when access is restricted [6]. The underlying mechanisms of social media addiction share similarities with those of other types of addiction. Excessive engagement with social networks may contribute to psychological problems, particularly among young people. The symptoms observed in individuals dependent on social media resemble those experienced by individuals with substance use disorders or other behavioural addictions [7, 8]. Common consequences of excessive social media use among adolescents include sleep disturbances, reduced attention span, negative self-perception, and cyberbullying [9, 10]. Pantic demonstrated that overuse of social networking sites is associated with increased depressive symptoms and negatively affects self-esteem [11].

Adolescence is a critical developmental period characterised by increased emotional reactivity and ongoing maturation of emotion regulation and self-control processes. During this period, adolescents’ psychological well-being plays a significant role in shaping behavioural patterns, including digital media use.

According to Self-Determination Theory, psychological well-being and happiness depend on the satisfaction of three basic psychological needs: autonomy, relatedness, and competence [12]. When these needs are insufficiently fulfilled, individuals may seek alternative sources of gratification to compensate for diminished well-being. Social media platforms may temporarily satisfy these needs by offering rapid feedback, social validation, and opportunities for interaction. However, reliance on such external and short-term sources of gratification may increase vulnerability to excessive and problematic social media use.

This perspective is complemented by Compensatory Internet Use Theory, which suggests that individuals may engage in excessive internet or social media use as a coping strategy to alleviate negative emotional states or psychological distress [13]. From this viewpoint, adolescents experiencing lower levels of happiness or psychological well-being may turn to social media as a means of emotional regulation or escape from unpleasant emotions. Although such use may initially provide relief, it may gradually evolve into uncontrolled and addictive patterns of behaviour.

Within this theoretical framework, happiness is considered a key psychosocial factor associated with social media addiction. Happiness is characterised by the predominance of positive emotions and overall satisfaction with life [14]. Several studies have reported that individuals with higher levels of happiness exhibit greater life satisfaction and that happiness may have a protective effect against the development of social media addiction [15, 16]. In contrast, individuals who feel unhappy may seek distractions to escape unpleasant emotions and may thus turn to social media as a coping mechanism. Although initially perceived as comforting, such use may progressively escalate to a point where the individual is unable to control it. Therefore, individuals with higher levels of happiness may have a lower risk of developing social media addiction.

Based on this theoretical background, this study aimed to determine the level of social media addiction among high school students, to examine factors associated with social media addiction, and to assess their subjective happiness levels.

## Methods

### Participants and Procedure

This cross-sectional study was conducted between February 5 and April 29, 2025, among students attending high schools in the study area. The data collection period was determined according to the school academic calendar, the timing of administrative permissions obtained from the participating schools, and the feasibility of accessing students during regular class schedules while avoiding examination periods. In total, 86 high schools were available, including 27 (31.4%) private and 59 (68.6%) public institutions. The total number of students enrolled in these schools was 35,605, of whom 6,107 (17.2%) attended private and 29,588 (82.8%) attended public high schools.

The required sample size for this study was calculated as 365 students (assuming 50% prevalence, 95% confidence interval, and 5% margin of error). Considering the design effect associated with the cluster sampling design, the initially calculated sample size was increased to 760 students. A cluster sampling design was used to ensure representativeness across different school types while maintaining feasibility within the school-based setting. Each high school was considered a cluster; one private and three public high schools were selected by simple random sampling. The number of students included from each school was proportionally weighted according to the total number of students in private and public high schools.

Parents were informed about the aim and scope of the study through school administrators, and written informed consent was obtained from parents who approved their child’s participation. Students were informed about the study procedures prior to data collection and provided assent before completing the questionnaire. Participation was voluntary and anonymous.

Data were collected in classroom settings during scheduled lesson hours under researcher supervision. Completion of the questionnaire required 10–15 min.

### Measures

A structured questionnaire was used to collect data, developed based on the study objectives and relevant literature [4, 5, 7]. The questionnaire included items on sociodemographic characteristics, variables presumed to be associated with social media addiction, the Social Media Addiction Scale for Adolescents (SMAS-A), and the Subjective Happiness Scale (SHS).

### Social Media Addiction Scale for Adolescents

Students’ levels of social media addiction were assessed using the Social Media Addiction Scale for Adolescents (SMAS-A). The scale was developed by Özgenel et al. in 2019 [17] and consists of nine items on a five-point Likert scale. Response options are scored as follows: “Never: 1 point,” “Rarely: 2 points,” “Sometimes: 3 points,” “Often: 4 points,” and “Always: 5 points.” Total scores range between 9 and 45, with higher scores indicating higher levels of social media addiction. In the present study, the internal consistency of the SMAS-A was acceptable (Cronbach’s α = 0.83).

### Subjective Happiness Scale

Students’ subjective happiness levels were assessed using the Subjective Happiness Scale (SHS). The scale was developed by Lyubomirsky and Lepper [18], and its Turkish validity and reliability study was conducted by Doğan and Totan [19]. The scale consists of four items on a seven-point Likert scale; the fourth item is reverse scored. Responses range from “1 = not very happy” to “7 = very happy.” Total scores range between 4 and 28, with higher scores indicating higher levels of subjective happiness. The SHS demonstrated a Cronbach’s alpha of 0.65, which is considered acceptable for brief measures.

### Statistical Analysis

Data were analyzed using the IBM SPSS Statistical Package Program, version 24.0 (IBM Corp., Armonk, NY, USA). Normality of distribution was assessed using the Kolmogorov–Smirnov test. As the distribution of the main variables deviated from normality, non-parametric tests were applied. Accordingly, the Mann–Whitney U test, Kruskal–Wallis test, and Spearman correlation analysis were used for statistical analyses.

Multiple linear regression analysis was performed to identify the factors associated with social media addiction. The assumptions of linear regression (linearity, multicollinearity, homoscedasticity, independence of errors, and normality of residuals) were examined prior to model estimation. The dependent variable was the total score of the SMAS-A. Seven independent variables (gender, place of residence, peer group composition, quality of friendship relationships, age at first social media use, daily duration of social media use, and SHS score) were entered into the model simultaneously. The model fit was evaluated using the F-statistic, and the proportion of explained variance was reported using the coefficient of determination (R²). Standardized regression coefficients (β) and 95% confidence intervals were presented for each predictor. A significance level of *p* < 0.05 was considered statistically significant.

### Ethical Considerations

Ethical approval was obtained from the relevant institutional ethics committee (Approval No: 79, Date: 11 February 2025). Written permission for data collection was granted by the city Provincial Directorate of National Education on 24 March 2025. Informed written assent was taken from the participants and informed written consent was obtained from their parents. The study was conducted in accordance with the ethical principles of the Declaration of Helsinki, International Council for Harmonisation Good Clinical Practice guidelines and applicable regulatory requirements.

## Results

### Sample Characteristics

Of the participants, 563 (65.6%) were female and 295 (34.4%) were male. Their ages ranged from 13 to 21 years, with a mean of 15.8 ± 1.1 years (median = 16.0). Among the students, 324 (37.8%) were in the ninth grade, while 87 (10.1%) were in the 12th grade. A total of 836 participants (97.4%) lived with their families, and 760 (88.6%) reported living in a nuclear family structure. Of the participants, 498 (58.0%) stated that their family’s income level was moderate (Table 1).

### Levels of Social Media Addiction and Subjective Happiness

Overall, students demonstrated a moderate level of social media addiction, with SMAS-A scores ranging from 9 to 45 and a median score of 20.0 (Q1–Q3: 16.0–25.0). Subjective happiness scores ranged from 4 to 28, with a median score of 18.0 (Q1–Q3: 14.0–21.0), indicating a mid-range level of happiness among the study population.

### Bivariate Analyses

In bivariate analyses, SMAS-A scores differed significantly according to gender, place of residence, family type, peer group composition, quality of friendship relationships, age at first social media use, and daily duration of social media use (Tables 1 and 2). No significant differences were observed with respect to grade level, age group, body mass index, family income status, parental education level, parental employment status, or possession of a personal digital device.

Variables that were significantly associated with social media addiction in bivariate analyses were further examined using multivariate linear regression analysis to identify independent predictors.

**Table 1:** Distribution of students’ SMAS-A scores according to sociodemographic characteristics

Characteristics	Frequency *n* (%)	SMAS-A Score Median (Q1​ - Q3​)	Statistical Analysis z/KW; *p*-value
*Grade level*			6.547; 0.088
9th grade	324 (37.8)	19.0 (15.0–24.0)	
10th grade	267 (31.1)	21.0 (16.0–26.0)	
11th grade	180 (21.0)	21.0 (16.0–25.0)	
12th grade	87 (10.1)	20.0 (15.0–24.0)	
*Age group*			1.440; 0.150
≤ 15	394 (45.9)	19.0 (15.0–24.0)	
≥ 16	464 (54.1)	21.0 (16.0–25.0)	
*Gender*			**5.301; <0.001**
Female	563 (65.6)	21.0 (16.0–26.0)	
Male	295 (34.4)	18.0 (15.0–22.0)	
*Body mass index (BMI)*			1.121; 0.571
≤ 18.49 (Underweight)	165 (19.2)	20.0 (16.0–25.0)	
18.50-24.99 (Normal weight)	567 (66.1)	20.0 (15.0–25.0)	
≥ 25.0 (Overweight/Obese)	126 (14.7)	20.0 (16.0–24.0)	
*Place of residence*			**2.812; 0.005**
Residing with family	836 (97.4)	20.0 (16.0–25.0)	
Residing elsewhere (Not with family)	22 (2.6)	18.0 (13.0–24.0)	
*Family type*			**2.801; 0.005**
Nuclear	760 (88.6)	20.0 (15.0–25.0)	
Extended	44 (5.1)	21.0 (17.0–27.0)	
Broken/Fragmented	54 (6.3)	21.0 (17.0–25.0)	
*Family income status*			1.268; 0.531
Poor	19 (2.2)	22.0 (18.0–34.0)	
Moderate	498 (58.1)	20.0 (16.0–25.0)	
Good	341 (39.7)	20.0 (15.0–24.0)	
*Mother’s education level*			0.209; 0.901
Secondary school and below	312 (36.4)	20.0 (15.0–25.5)	
High school	261 (30.4)	20.0 (16.0–24.0)	
University/College	285 (33.2)	20.0 (16.0–25.0)	
*Father’s education level*			1.980; 0.372
Secondary school and below	178 (20.8)	20.0 (16.0–25.0)	
High school	340 (39.6)	21.0 (15.0–25.5)	
University/College	340 (39.6)	19.5 (15.0–24.0)	
*Mother’s current employment status*			0.695; 0.487
Unemployed	495 (57.7)	20.0 (16.0–25.0)	
Employed	363 (42.3)	20.0 (15.0–24.0)	
*Father’s current employment status*			1.538; 0.124
Unemployed	82 (9.6)	21.0 (17.0–27.0)	
Employed	776 (90.4)	20.0 (15.0–25.0)	
*Total*	858 (100.0)	20.0 (16.0–25.0)	-

z = Mann–Whitney U test; KW = Kruskal–Wallis test; *p* < 0.05 was considered statistically significant

Regarding characteristics potentially associated with social media addiction, the study population showed variability in academic performance, peer group composition, friendship quality, age at first social media use, and daily duration of social media use. The distribution of SMAS-A scores according to these characteristics pesented in Table 2

**Table 2:** Distribution of SMAS-A scores according to selected characteristics related to social media addiction

Characteristics	Frequency *n* (%)	SMAS-A Score Median (Q1​ - Q3​)	Statistical Analysis z/KW; *p*-value
*End-of-term academic grade point average (Score)*			0.197; 0.906
≤ 69	140 (16.3)	21.0 (16.0–25.0)	
70–84	247 (28.8)	20.0 (15.0–25.0)	
≥ 85	471 (54.9)	20.0 (16.0–25.0)	
*Composition of peer group in school environment*			**20.903; <0.001**
Male	189 (22.0)	18.0 (15.0–22.0)	
Female	400 (46.6)	21.0 (17.0–26.0)	
Equal number of males and females	269 (31.4)	20.0 (15.0–25.0)	
*Description of friendship relationships*			**7.113; 0.029**
Poor	29 (3.4)	26.0 (16.0–36.0)	
Moderate	202 (23.5)	21.0 (16.0–25.0)	
Good	627 (73.1)	20.0 (15.0–24.0)	
*Possession of own mobile phone*,* tablet*,* or computer*			0.909; 0.363
No	28 (3.3)	21.0 (15.5–28.5)	
Yes	830 (96.7)	20.0 (16.0–25.0)	
*Age of first social media use*			**9.365; 0.009**
≤ 10	245 (28.6)	21.0 (16.0–26.0)	
11–14	567 (66.1)	20.0 (16.0–25.0)	
≥ 15	46 (5.4)	17.5 (11.0–23.0)	
*Average daily time spent on social media (hours)*			**102.451; <0.001**
≤ 2	197 (23.0)	16.0 (13.0–20.0)	
3–4	340 (39.6)	20.0 (15.0–24.0)	
≥ 5	321 (37.4)	23.0 (18.0–28.0)	
*Total*	858 (100.0)	20.0 (16.0–25.0)	-

z = Mann–Whitney U test; KW = Kruskal–Wallis test; *p* < 0.05 was considered statistically significant

**Fig. 1:**
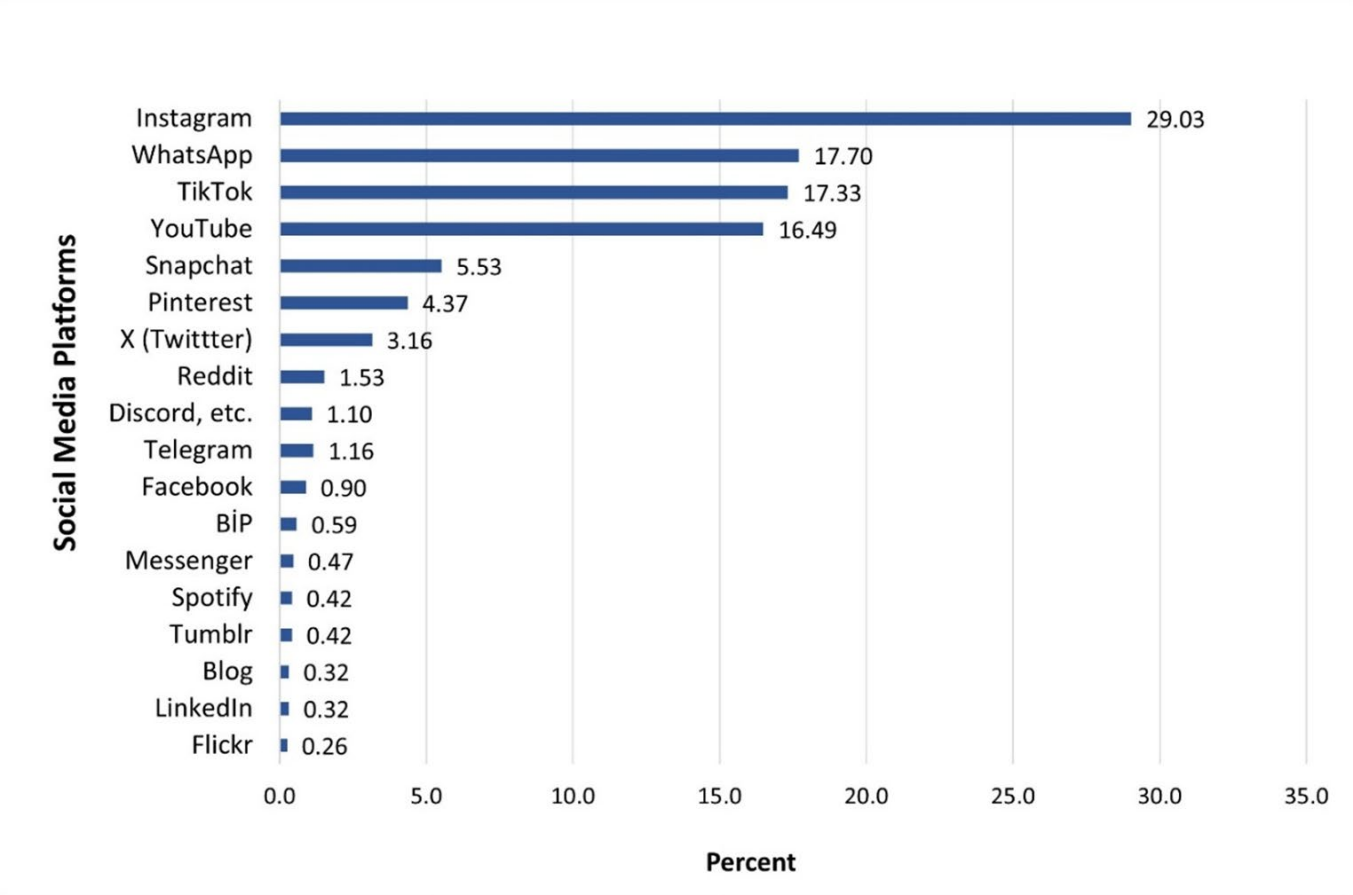
Distribution of social media platforms used by the study participants. *The figures (percentages) were evaluated based on the responses provided, not the individuals

**Fig. 2:**
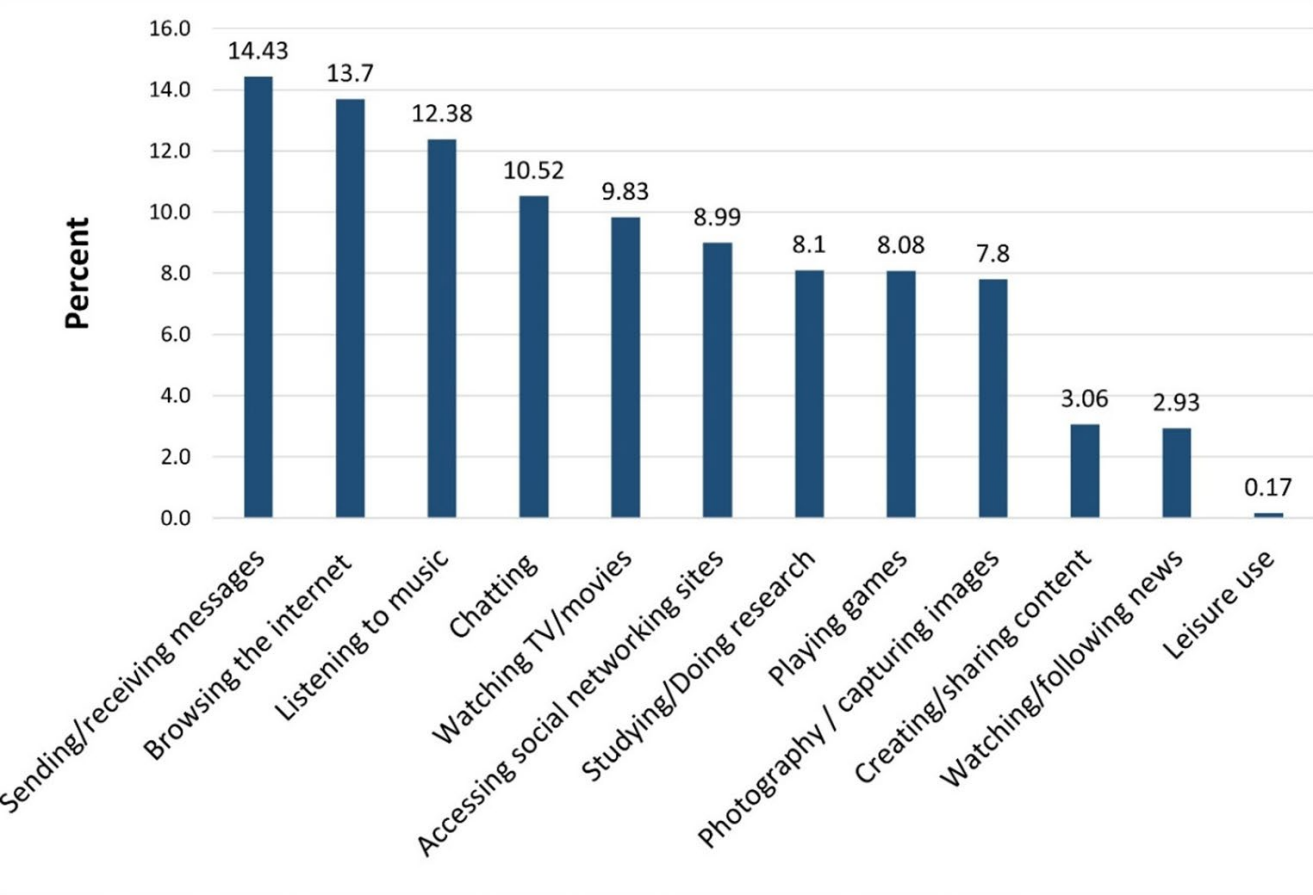
Percentage distribution of internet/social media usage purposes of the study group. *The figures (percentages) were evaluated based on the responses provided, not the individuals

Instagram, WhatsApp, and TikTok were the most frequently used platforms, while communication, general browsing, and music consumption were the primary usage purposes (Figs. 1 and 2).

The Multiple Linear Regression Analysis (Table 3), conducted to predict scores on the SMAS-A, revealed that the overall model was statistically significant (F = 22.410; *p* < 0.001). The seven independent variables included in the model explained 15.6% of the total variance in social media addiction scores (R^2^ = 0.156). Examination of the individual predictor variables showed that gender (β = -0.149, *p* < 0.001), friendship relationship (β = 0.066, *p* = 0.043), age of first social media use (β = 0.086, *p* = 0.007), daily social media use duration (β = 0.290, *p* < 0.001), and Subjective Happiness Scale score (β = -0.129, *p* < 0.001) were all statistically significant predictors of social media addiction. Specifically, a longer daily social media use duration was the strongest positive predictor (β = 0.290), indicating that increased duration is associated with higher addiction scores. Conversely, being female and higher SHS scores were associated with lower social media addiction.

**Table 3:** Results of multiple linear regression analysis of variables associated with social media addiction (*N* = 858)

Variables	B	95% CI [Lower-Upper]	β	*p*
Constant	19.843	[17.545, 22.142]	-	**< 0.001**
Gender	-2.212	[-3.218, -1.207]	-0.149	**< 0.001**
Place of residence	-1.795	[-4.569, 0.979]	-0.040	0.204
Peer group composition	-0.330	[-0.986, 0.327]	-0.034	0.325
Friendship relationship	0.878	[0.028, 1.727]	0.066	**0.043**
Age of first SM use	1.143	[0.310, 1.977]	0.086	**0.007**
Daily SM use duration	0.876	[0.685, 1.067]	0.290	**< 0.001**
Subjective Happiness Scale	-0.188	[-0.280, -0.095]	-0.129	**< 0.001**
F = 22.410; *p* < 0.001. R^2^ = 0.156				

SM = social media; B = Unstandardized Coefficient; 95% CI: 95% Confidence Interval; β = Standardized Coefficient

## Discussion

Numerous prevalence studies have been conducted worldwide on social media addiction, which has emerged as a growing public health concern. In a meta-analysis conducted by Cheng et al. [20], the prevalence of social media addiction was reported to range from 0.0% to 83.0%, with an average prevalence of 24.0%. Similarly, Salari et al. [21] reported prevalence rates ranging between 0.5% and 73.0%, with an overall estimate of 18.4%. In the present study, based on the scores obtained from the SMAS-A, students demonstrated a moderate level of social media addiction. Consistent with these findings, Colonio [22] reported moderate-to-high risk levels among adolescents, whereas Arslan et al. [23] observed lower levels. Although direct prevalence comparisons are limited due to different measurement tools and cut-off criteria, the observed levels suggest that problematic social media use represents a meaningful mental health concern within this adolescent population. Variations across studies may reflect sociocultural context, digital accessibility, and differences in psychosocial vulnerability.

Social media addiction levels were higher among female students, and gender remained a significant predictor in the multivariate model. This finding aligns with previous research suggesting that females may be more susceptible to social media addiction due to greater involvement in social networking activities, social approval seeking, and emotion-focused use patterns [23, 24]. However, inconsistent findings in the literature indicate that gender effects may vary across populations and usage motivations [25, 26].

Peer relationships emerged as an independent predictor of social media addiction. Students reporting poorer friendship relationships had higher addiction scores. This finding is consistent with previous research emphasizing the importance of peer dynamics in adolescent well-being [27]. Beyens et al. [28] demonstrated that the emotional impact of social media use varies depending on usage patterns. Adolescents experiencing interpersonal dissatisfaction may turn to online environments as a compensatory coping mechanism [13]. While social media may temporarily provide social validation and belonging, repeated reliance on digital interaction may reinforce maladaptive behavioural patterns.

According to the 2024 Children’s Information and Communication Technology Usage Survey, 66.1% of children use social media, with usage rates increasing markedly with age [29]. Early access to digital devices facilitates exposure to social media even before the age restrictions of 13 years defined by platforms [30]. Such early exposure may normalize intensive digital engagement during critical developmental periods.

Consistent with this contextual background, age at first social media use was a significant predictor in the present study. Students who initiated social media use at younger ages demonstrated higher addiction levels. Early exposure may increase susceptibility to reinforcement-based digital behaviours before the full maturation of self-regulation capacities. These findings are consistent with Stănculescu and Griffiths [31], who identified early initiation as a risk factor for addiction-like profiles. Given that adolescence is marked by heightened emotional reactivity and ongoing executive function development, early exposure may amplify vulnerability to compulsive use patterns [32].

Daily duration of social media use was the strongest predictor in the regression model. Spending five hours or more per day on social media was associated with higher addiction scores. Similar findings have been reported by Sönmez Sarı et al. [33]. Prolonged exposure to social media has been linked to depressive symptoms, sleep disturbances, and emotional dysregulation [34, 35]. Although duration alone does not define addiction, excessive time investment may reflect impaired control and behavioural preoccupation.

Subjective happiness was negatively associated with social media addiction, although the effect size was modest. This finding aligns with studies reporting a protective role of well-being against problematic internet use [15, 16]. According to Self-Determination Theory [12], unmet psychological needs may lead individuals to seek external sources of gratification. Social media platforms may temporarily fulfil needs for relatedness and validation; however, reliance on such external reinforcement may not produce sustained well-being. The modest effect size suggests that happiness is one of multiple psychosocial factors influencing addiction risk, rather than a dominant determinant. Previous studies have also reported mixed associations between social media use and happiness [36, 37], indicating a complex and bidirectional relationship.

### Limitations

The cross-sectional design of the study, the lack of objective measurement tools for assessing social media addiction and subjective happiness, and the exclusion of high schools located in rural areas constitute the main limitations of this study.

## Conclusion

The findings of this study suggest that students have a moderate level of social media addiction. Gender, family structure, quality of relationships with friends, age at first social media use, average daily time spent on social media, and subjective happiness were identified as predictors of social media addiction. A weak negative association was observed between subjective happiness and social media addiction. Limiting social media use may help prevent the development of addiction; therefore, awareness-raising activities targeting both students and parents are recommended. Further comprehensive studies are needed to better elucidate the relationship between social media addiction and subjective happiness.
